# NOP2 facilitates EZH2-mediated epithelial–mesenchymal transition by enhancing EZH2 mRNA stability via m5C methylation in lung cancer progression

**DOI:** 10.1038/s41419-024-06899-w

**Published:** 2024-07-16

**Authors:** Ying Yang, Hongzhao Fan, Hongyang Liu, Xueling Lou, Nan Xiao, Chenxing Zhang, Huanxiang Chen, Shuangshuang Chen, Huihui Gu, Hongchun Liu, Junhu Wan

**Affiliations:** 1 Department of Clinical Laboratory, The First Affiliated Hospital of Zhengzhou University, Zhengzhou, Henan China; 2 Kidney Transplantation Unit, The First Affiliated Hospital of Zhengzhou University, Zhengzhou, Henan China; 3 Department of Obstetrics and Gynecology, The Third Affiliated Hospital of Zhengzhou University, Zhengzhou, Henan China; 4 School of Life Science, Zhengzhou University, Zhengzhou, Henan China; 5 The Second Clinical Medical College of Henan University of Traditional Chinese Medicine, Zhengzhou, Henan China

**Keywords:** Lung cancer, Biomarkers

## Abstract

NOP2, a member of the NOL1/NOP2/SUN domain (NSUN) family, is responsible for catalyzing the posttranscriptional modification of RNA through 5-methylcytosine (m5C). Dysregulation of m5C modification has been linked to the pathogenesis of various malignant tumors. Herein, we investigated the expression of NOP2 in lung adenocarcinoma (LUAD) tissues and cells, and found that it was significantly upregulated. Moreover, lentivirus-mediated overexpression of NOP2 in vitro resulted in enhanced migration and invasion capabilities of lung cancer cells, while in vivo experiments demonstrated its ability to promote the growth and metastasis of xenograft tumors. In contrast, knockdown of NOP2 effectively inhibited the growth and metastasis of lung cancer cells. RNA-sequencing was conducted to ascertain the downstream targets of NOP2, and the findings revealed a significant upregulation in EZH2 mRNA expression upon overexpression of NOP2. Subsequent validation experiments demonstrated that NOP2 exerted an m5C-dependent influence on the stability of EZH2 mRNA. Additionally, our investigations revealed a co-regulatory relationship between NOP2 and the m5C reader protein ALYREF in modulating the stability of EZH2 mRNA. Notably, the NOP2/EZH2 axis facilitated the malignant phenotype of lung cancer cells by inducing epithelial**–**mesenchymal transition (EMT) both in vitro and in vivo. Mechanistically, ChIP analysis proved that EZH2 counteracted the impact of NOP2 on the occupancy capacity of EZH2 and H3K27me3 in the promoter regions of E-cadherin, a gene crucial for regulating EMT. In a word, our research highlights the significant role of NOP2 in LUAD and offers novel mechanistic insights into the NOP2/ALYREF/EZH2 axis, which holds promise as a potential target for lung cancer therapy.

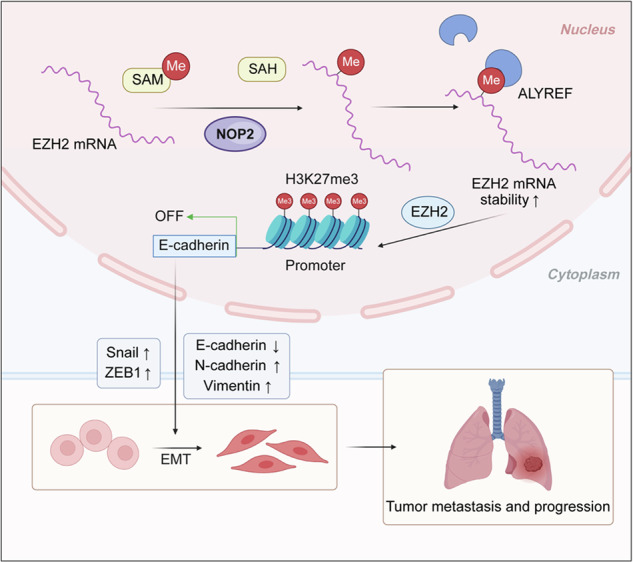

## Introduction

Lung cancer, a malignant neoplasm originating in the bronchial mucosa or glands of the lungs, exhibits a high and increasing incidence globally. The pathohistological classification of lung cancer primarily encompasses two major categories, namely non-small cell lung cancer (NSCLC) and small cell lung cancer (SCLC), and treatment decisions are contingent upon the specific subtype and stage of the cancer [1]. NSCLC, which accounts for 85% of all lung cancers, is further subdivided into squamous-cell carcinoma, adenocarcinoma, and large-cell carcinoma [2]. Among these, lung adenocarcinoma (LUAD) is the prevailing form of lung cancer, accounting for approximately 40% of all lung cancer cases [3]. Surgical intervention is the primary approach for managing early-stage lung cancer, whereas advanced and metastatic cases necessitate systemic therapy [4, 5]. These treatments can significantly improve patients’ symptoms and extend their overall survival. However, the therapeutic efficacy in lung cancer patients is frequently constrained by drug resistance and the evasion of apoptosis by tumor cells, culminating in tumor recurrence and unfavorable prognoses [6]. Therefore, it is imperative to investigate the molecular mechanisms and potential therapeutic targets associated with lung cancer.

The m5C methylation occurs at the fifth position of cytosine residues in DNA and RNA, which is catalyzed by m5C methyltransferases with S-adenosine-L-methionine (SAM) as the methyl donor [7]. Similar to m6A methylation, the enzymes that regulate m5C RNA levels can be functionally categorized as “writers,” “erasers,” and “readers.” Methyltransferases, the “writers,” are responsible for transferring methyl to cytosine, and they primarily consist of seven members of the NOL1/NOP2/SUN domain (NSUN) family (NSUN1–7), and DNA methyltransferase analog 2 (DNMT2). “Erasers,” which remove m5C modifications from RNA, have been identified as alpha-ketoglutarate–dependent dioxygenase ABH1 (ALKBH1) and ten-eleven translocation family proteins (TET) [8, 9]. In addition, RNA m5C-binding proteins, such as Aly/REF export factor (ALYREF) and Y-box–binding protein 1 (YBX1), exert biological effects by recognizing and binding to m5C sites [10, 11]. The widespread presence of m5C modifications in RNA serves as an epigenetic alteration in tumorigenesis, and exerts significant influence on various physiological and pathological processes such as tumorigenesis, tumor cell migration, viral replication, stress response, and embryogenesis [12]. Methyltransferases in m5C regulate substrate levels by catalyzing m5C modifications in target RNAs, mediating cross-linking of a series of oncogenic or anti-tumorigenic factors, thereby affecting tumorigenesis and cancer progression. Therefore, abnormalities in m5C modification may become biomarkers for some tumors and are expected to be applied in early diagnosis and treatment of cancer. Notably, the relationship between m5C modification and cancer is still under intensive study, and its specific mechanism and role need to be further clarified.

As a member of the NSUN family of m5C methyltransferases, NOP2 (NSUN1, nucleolar protein p120) is mainly localized in the nucleus. Early studies have demonstrated that NOP2 plays a crucial role in catalyzing m5C methylation in yeast, particularly at the C2870 site of 25s rRNA, thereby influencing the maturation of 60s rRNA [13]. Human NOP2 binds to the 5’ETS region of pre-rRNA transcripts and regulates pre-rRNA processing by forming a noncatalytic complex with box C/D snoRNA [14]. In HIV-1 virus, NOP2 interacts with HIV-1 TAR RNA at the 5’-long terminal repeat sequence (LTR) by competing with the Tat protein and promotes TAR m5C methylation, thereby inhibiting viral transcription and promoting viral latency time [15]. NOP2 exhibits aberrant expression in a variety of cancers, such as renal clear-cell carcinoma, breast cancer, prostate cancer, and lung cancer, which is associated with poor prognosis [16–19]. However, the functional and molecular mechanisms by which NOP2 regulates lung cancer are still largely elusive.

Here, we conducted a series of in vitro and in vivo experiments to systematically characterize the biological role of NOP2 and its possible molecular mechanisms in lung cancer. The results revealed a significant upregulation of NOP2 in both lung cancer cells and tissues, and a higher expression of NOP2 was indicative of a poorer prognosis in lung cancer patients. In addition, NOP2 was found to play a crucial role in promoting lung cancer progression by enhancing the stability of EZH2 mRNA in an m5C-dependent manner, thereby facilitating epithelial–mesenchymal transition (EMT) in lung cancer cells. Our findings offer new perspectives on the molecular mechanisms of epigenetic alterations in lung cancer carcinogenesis and suggest that NOP2 may be a potential therapeutic target in lung cancer.

## Materials and methods

### Bioinformatics analysis

The bioinformatics tools utilized in this study are listed in Table S1.

### Patients and clinical samples

This study utilized pathological tissues obtained from the First Affiliated Hospital of Zhengzhou University, comprising cancerous tissues and adjacent normal tissues from 43 patients diagnosed with lung adenocarcinoma. The patient inclusion criteria were: (1) histopathological confirmation of lung adenocarcinoma; (2) no prior exposure to radiotherapy or chemotherapy before surgery; (3) absence of significant perioperative complications; and (4) no concurrent cancers other than lung adenocarcinoma. Following extraction, each tissue sample was promptly cryopreserved in liquid nitrogen. Informed consent was obtained from each participant, and the research protocol was approved by the Ethics Committee of the First Affiliated Hospital of Zhengzhou University.

### Immunohistochemical (IHC) staining

Prepared paraffin-embedded tissue slides underwent pre-baking, followed by dewaxing using xylene and hydration with varying concentrations of ethanol. Subsequently, the slides were subjected to antigen retrieval by heating in citrate buffer (0.01 mol/L, pH 6.0). To eliminate endogenous peroxidase activity, a solution of 3% H_2_O_2_ in deionized water was applied, followed by blocking with goat serum for 20 min. The primary antibody was introduced and left to incubate overnight at 4 °C. The following day, sections were triple-washed with phosphate-buffered saline (PBS), and an enzyme-labeled secondary antibody (PV-6000 goat anti-rabbit IgG/HRP polymer) was added, incubating at room temperature for 1 h. Subsequently, the sections were stained according to the instructions provided in the DAB kit. Nuclei were counterstained with hematoxylin, subjected to dehydration using gradient ethanol, cleared with xylene, and finally sealed with neutral gum. The IHC results were analyzed and scored by two pathologists without knowing the patients’ clinical information.

### Cell culture

The A549 cells and BEAS-2B cells were cultured in DMEM (Gibco, USA). The H358, H1650, H1299 cells were cultured in RPMI1640 (Gibco, USA). All cells were cultured at 37 °C with 5% CO_2_ and routinely supplemented with 10% fetal bovine serum (FBS) (Clark, USA) and 1% penicillin and streptomycin. All experiments were conducted using mycoplasma-free cells. BEAS-2B cells were acquired from Servicebio Biotechnology Co., Ltd., while other cell lines were obtained from Procell Life Science & Technology Co., Ltd. All cell lines were authenticated via short tandem repeat (STR) profiling.

### Western blot

Tissues and cells underwent lysis using ice-cold RIPA lysis buffer (Beyotime Biotechnology, China) supplemented with protease inhibitors. Following quality control, protein samples were separated through 8% or 10% Bis-Tris SDS-PAGE gel and subsequently transferred onto polyvinylidene fluoride (PVDF) membranes. The membranes were then subjected to overnight incubation with primary antibodies at 4 °C, followed by incubation with the respective horseradish peroxidase-conjugated secondary antibody. Signal detection was accomplished through chemiluminescence (GE Amersham Imager600, USA). The primary antibodies utilized in this study are listed below: anti-NOP2 (Proteintech, 10448-1-AP), anti-β-Actin (Beyotime, AA128), anti-EZH2 (Cell Signaling Technology, #5246), anti-ALYREF (Abways, CY8314), anti-Snail (Beyotime, AF8013), anti-ZEB1 (Beyotime, AF8388), anti-E-cadherin (Abways, AY9249), anti-N-cadherin (Abways, CY5015), anti-Vimentin (Abways, CY5134), anti-H3K27me3 (Cell Signaling Technology, # 9733).

### Quantitative real-time PCR (RT-qPCR)

Total RNA was extracted with the RNA Extraction Trizol Kit (Takara, China). The RNA was reverse-transcribed into cDNA by Hifair III 1st Strand cDNA Synthesis SuperMix for qPCR (Yeasen, China) and then subjected to an RT-qPCR assay using Hieff qPCR SYBR Green Master Mix (Low Rox) (Yeasen, China). Relative mRNA expression was determined using the 2^-ΔΔCt^ method, and GAPDH was applied as the internal control for normalization. The primer sequences are listed in Table S2.

### Lentiviral transduction and cell transfection

Lentivirus constructs were purchased from Genechem (Shanghai, China). 2 × 10^5^ cells were cultured in a six-well plate and then transfected with NOP2 or EZH2 overexpression (i.e., NOP2 or EZH2) or knockdown (sh-NOP2, sh-EZH2, sh-ALYREF) recombinant lentivirus after 24 h. Subsequently, lung cancer cells were cultured in medium containing 3ug/mL puromycin for 72 h to select stably transfected cells for next studies. Human overexpression of ALYREF plasmids were purchased from Miaoling Biology (Wuhan, China). Transient transfection was performed using Lipofectamine 3000 (Invitrogen, USA) according to the manufacturer’s instructions. After 48 h of culture, cells were harvested for western blot.

### Transwell and wound-healing assays

Transwell chambers (Corning, USA) were employed for the conducted assays. In invasion assays, Matrigel (Yeasen, China) was thawed overnight at 4 °C. Add 60 µL of Matrigel mixture diluted in serum-free medium to the upper chamber and incubate at 37 °C for 30 min to solidify. Subsequently, 5 × 10^4^ cells in 200 µL serum-free medium were placed in the upper chamber, and 500 µL of 20% FBS-supplemented medium was added to the lower chamber. After a 12-h incubation at 37 °C, both chambers were immersed in 10% formaldehyde and stained with 0.1% crystal violet for 20 min. The cells in the upper chamber were then wiped off with cotton swabs, and the stained cells on the surface of the lower membrane were counted under the microscope. The transwell migration experiment followed the same procedures as the invasion assays, except that Matrigel was omitted in the upper chambers.

For the wound-healing assay, lung cancer cells were plated in 6-well plates. Start scratched when the cell confluence reached 90%. Then add medium containing 1% FBS and continue to incubate at 37 °C in a 5% CO_2_ incubator. The healing of the scratches was recorded at 0 and 24 or 48 h.

### Cell Counting Kit-8 (CCK-8) and colony-formation assay

The proliferation capacity of lung cancer cells was assessed through a CCK-8 assay. Cells were plated at a density of 1000 cells/well in 96-well plates and incubated in a 5% CO_2_ atmosphere at 37 °C. CCK-8 reagent (Beyotime Biotechnology, China) was introduced at 24, 48,72, 96, and 120 h post-seeding, and the absorbance at 450 nm was measured two hours after incubation.

For the colony-formation assay, cells were cultivated in six-well plates with 1000 cells per well and maintained in a 5% CO_2_ atmosphere at 37 °C for 10–14 days. Following paraformaldehyde fixation and crystal violet staining, the count of formed colonies was determined.

### EdU staining assessment

The EdU staining assay was conducted using the EdU assay kit (Beyotime Biotechnology, China). In brief, cells were treated with 10 μM EdU for 2 h, fixed with 4% paraformaldehyde, and permeabilized with 0.5% Triton X-100. Subsequently, the treated cells were incubated with Click Reaction Solution, consisting of Click Additive Solution, CuSO_4_, and Azide 488, for 30 min at room temperature, shielded from light. Following this, the samples were stained with Hoechst 33342 for 10 min. Images were captured using a fluorescence microscope (Olympus, Japan) to quantify the proportion of EdU-positive cells.

### Flow cytometry

Cell apoptosis was assessed using the Annexin V-FITC Apoptosis Detection Kit (Beyotime Biotechnology, China). Concurrently, the cell cycle was analyzed with the PI Cell Cycle and Apoptosis Analysis Kit (Beyotime Biotechnology, China). Cells were cultured in 6-well plates, harvested following appropriate treatments, and stained with Annexin V-FITC and PI in accordance with the manufacturer’s instructions for apoptosis and cell cycle analysis. Flow cytometry was performed using a DxFLEX flow cytometer (Beckman Coulter, USA), and the obtained data were analyzed using FlowJo software.

### RNA-sequencing and analysis

Total RNAs were extracted from H1650 cells with stable NOP2 overexpression and vector-transfected cells using RNAiso Plus (Takara, Japan). Each group contains 3 parallel samples. RNA quality was determined by examining A260/A280 with NanodropTM OneC spectrophotometer (Thermo Fisher Scientific Inc, USA). RNA Integrity was confirmed by 1.5% agarose gel electrophoresis. Qualified RNAs were finally quantified by Qubit3.0 with QubitTM RNA Broad Range Assay kit (Life Technologies, Q10210). RNA-sequencing experiment and high through-put sequencing were conducted by Seqhealth Technology Co., Ltd. (Wuhan, China). 2 μg total RNAs were used for stranded RNA-sequencing library preparation using KC^TM^ Stranded mRNA Library Prep Kit for Illumina^®^ (Catalog NO. DR08402, Wuhan Seqhealth Co., Ltd. China) following the manufacturer’s instruction. PCR products corresponding to 200–500 bps were enriched, quantified and finally sequenced on DNBSEQ-T7 sequencer (MGI Tech Co., Ltd. China) with PE150 model.

Genes differentially expressed between groups were identified using DESeq2 R package (version 3.0.3), with a significance threshold of *p*-value ≤ 0.05 and |log_2_FoldChange| ≥ 1. Gene ontology (GO) analysis and Kyoto encyclopedia of genes and genomes (KEGG) enrichment analysis for differentially expressed genes were both implemented by the cluster Profiler R package (version 3.0.3) with a *p*-value cutoff of 0.05 to generate the enrichment bubble charts.

### RNA immunoprecipitation (RIP) and methylated RNA immunoprecipitation (MeRIP)

The interaction between NOP2 and ALYREF with EZH2 was detected by RNA immunoprecipitation kit (Geneseed, Guangzhou, China). In brief, 2 × 10^7^ cells were lysed by RIP lysis buffer and then incubated with magnetic beads conjugated with anti-IgG (Abcam, ab172730), NOP2 or ALYREF antibodies. The coprecipitated RNAs were examined using RT-qPCR. IgG was used as a negative control to preclude nonspecific binding. Similarly, MeRIP experiments were performed using the m5C MeRIP kit from CloudSeq Biotech Inc. (Shanghai, China), followed by RT-qPCR analysis of the extracted RNA.

### Luciferase reporter assay

Luciferase reporter plasmids containing either the wild-type EZH2 5’UTR or the mutant EZH2 5’UTR were synthesized by Genechem (Shanghai, China) and inserted into the GV272 vector. In 96-well plates, NOP2 knockdown cells were seeded and transfected with 200 ng of luciferase reporter plasmids and 10 ng of Renilla luciferase control reporter vectors. After 48 h, cells were lysed and analyzed for luciferase activity using a Dual Luciferase Reporter Assay Kit (E1910, Promega). The firefly luciferase activity was quantified with the EnVision® multimode plate reader (PerkinElemer) and normalized to the renilla luciferase activity.

### mRNA stability assay

To assess the mRNA stability in different lung cancer cell lines, we introduced actinomycin D (MedChemExpress, USA) at a concentration of 5 μg/mL. Subsequent to incubation for specified durations, cells were collected, and total RNA was extracted for RT-qPCR analysis to evaluate stability. The mRNA levels were normalized to the expression at 0 h.

### Protein stability assay

To determine protein stability, cells were treated with cycloheximide (MedChemExpress, USA) at a final concentration of 100 μg/mL for the indicated times. The expression of EZH2 was measured through western blot analysis.

### Protein-translation assay

In vitro assessment of protein-translation levels was conducted using puromycin (Solarbio, China). Cells were exposed to 200 ng/mL puromycin for the specified durations. Following treatment, cells were lysed, and protein expression was analyzed by western blot, with β-Actin serving as the reference.

### Immunofluorescence (IF) staining

Cells were grown on coverslips in a 24‐well plate, fixed with 4% paraformaldehyde for 15 min, permeabilized with 1% Triton X-100 for 15 min, and rinsed with PBS. After blocking, the cells were incubated with primary antibodies overnight at 4 °C. The following day, fluorescent secondary antibodies were applied and allowed to incubate on a shaker in the dark for 1 h. Cell nuclei were stained with 4′,6‐diamidino‐2‐phenylindole (DAPI). Immunofluorescence was visualized with a fluorescence microscope (Olympus, Japan).

### Animal experiments

Female BALB/c nude mice (4 weeks old) were purchased from GemPharmatech (Jiangsu, China) and raised under pathogen-free conditions. After a 5-day observation and acclimatization period, the mice were randomly assigned to experimental groups. For the subcutaneous transplantation model, mice received subcutaneous injections of 5×10^6^ stably transfected H1299 cells, which were first diluted in 200 μL of PBS. The tumor volume was observed and recorded once per week and the width (W) and length (L) of each tumor were measured with calipers using the following formula: *V* = (*L* × *W*^2^)/2. Mice were euthanized at predetermined time points, ensuring that no tumor exceeded a volume of 2000 mm³. Tumors were subsequently collected for further analysis. For the establishment of the lung metastasis model, 2 × 10^6^ stably transfected H1299 cells suspended in 100 μL of PBS were intravenously injected via the tail vein of BALB/c nude mice. Mice were euthanized 8 weeks post injection, and subsequent analysis was conducted on the metastatic lung tumors. All experimental animal procedures were approved by the Zhengzhou University Animal Care and Use Committee and performed in accordance with the Zhengzhou University Laboratory Animal Center Guidelines.

### Statistical analysis

The experimental data were analyzed utilizing SPSS 25.0 software and GraphPad Prism 8. All experiments were repeated at least three times, with results presented as the mean ± standard error of the mean (SEM). Statistical analyses were performed accordingly: the two-tailed independent samples *t*-test was employed for normally distributed data from two independent samples; conversely, the rank sum test (Mann–Whitney *U*-test) was utilized when the data did not adhere to a normal distribution. For comparing multiple sets of quantitative data, one-way analysis of variance (ANOVA) was used. A significance level of *p* < 0.05 was considered statistically significant.

## Results

### NOP2 is upregulated in lung cancer tissues and predicts poor prognosis

**Fig. 1:**
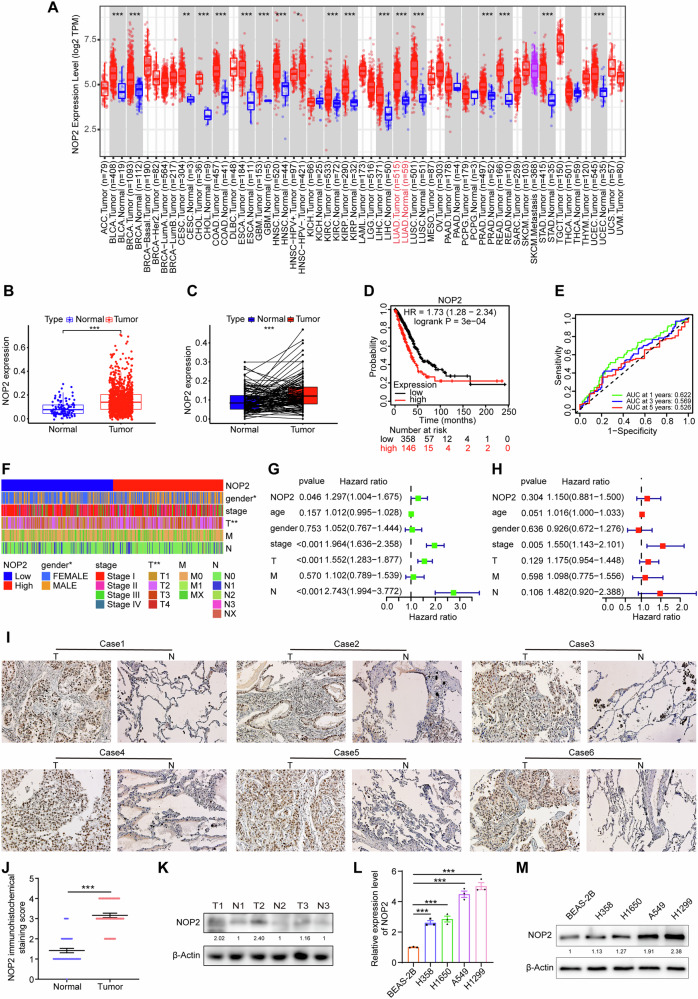
NOP2 is upregulated in lung cancer tissues and predicts poor prognosis. **A** TIMER 2.0 database was used for pan‐cancer analysis of NOP2. **B**, **C** Differential analysis of NOP2 expression in LUAD and normal tissues. **D**, **E** Kaplan–Meier analysis and receiver operating characteristic curve of NOP2. **F** The heat map of the relationship between NOP2 expression and different clinicopathologic features of LUAD. **G**, **H** Forest plots of cox regression analysis of the relationship between NOP2 expression and different clinicopathologic features of LUAD. **I** Representative IHC staining images and **J** IHC scores of NOP2 expression in paired LUAD tissues and adjacent non-tumor tissues. Scale bar = 50 μm. **K** Western blot for NOP2 expression in several specimens from LUAD patients. **L**, **M** RT-qPCR and western blot were used to compare the difference in NOP2 expression in lung cancer cells and normal human lung epithelial cells. Statistical methods: independent samples *t*-test (**J**), one-way ANOVA (**L**). Data are presented as the mean ± SEM of at least 3 independent experiments. *p < 0.05, **p < 0.01, ***p < 0.001.

To identify the cancer-related m5C genes in lung cancer, we first analyzed expression levels of all m5C genes using RNA-sequencing data from the TCGA dataset. Relevant bioinformatics analysis of NOP2 was performed using the TIMER 2.0 database, which showed that the expression of NOP2 was elevated in most of the tumors (Fig. 1A). Differential expression analysis indicated a significantly higher expression of NOP2 in LUAD than in normal tissues (Fig. 1B, C). In addition, survival analysis suggested that higher NOP2 levels were associated with poor prognosis of LUAD and that NOP2 had some diagnostic value for LUAD (Fig. 1D, E). To determine the association between NOP2 expression and clinicopathological features of LUAD, we conducted Spearman’s correlation analysis of NOP2 expression with age, gender, stage, T, N, and M classification. Heat maps and forest plots (Fig. 1F–H) demonstrated a potential correlation between NOP2 expression in LUAD and both gender and tumor stage. To further validate these findings, IHC staining was performed on 43 paired LUAD tissues and adjacent normal tissues collected from the First Affiliated Hospital of Zhengzhou University. In accordance with the aforementioned data, aberrant overexpression of NOP2 was found in LUAD tissues (Fig. 1I, J). Moreover, we verified the expression of NOP2 in several of these pairs of tissues using western blot assays (Fig. 1K). Subsequently, an extensive investigation was conducted to assess the expression of NOP2 at both the transcript and protein levels in human lung cancer cell lines and normal lung samples. Specifically, a range of lung cancer cell lines were scrutinized to determine the abundance of NOP2 mRNA, as shown in Fig. 1L. The results revealed that the majority of lung cancer cell lines exhibited elevated levels of NOP2 mRNA compared with normal human epithelial cells BEAS-2B. NOP2 expression was further evaluated in four distinct lung cancer cell lines through immunoblotting. The endogenous expression of NOP2 was significantly upregulated in two of the cell lines (A549 and H1299), whereas the other two lines (H358 and H1650) showed relatively low levels of endogenous NOP2 (Fig. 1M). In summary, these findings collectively suggest an upregulation of NOP2 in lung cancer, and elevated NOP2 expression is associated with a poorer prognosis.

### NOP2 promotes migration and invasion of lung cancer cells

**Fig. 2:**
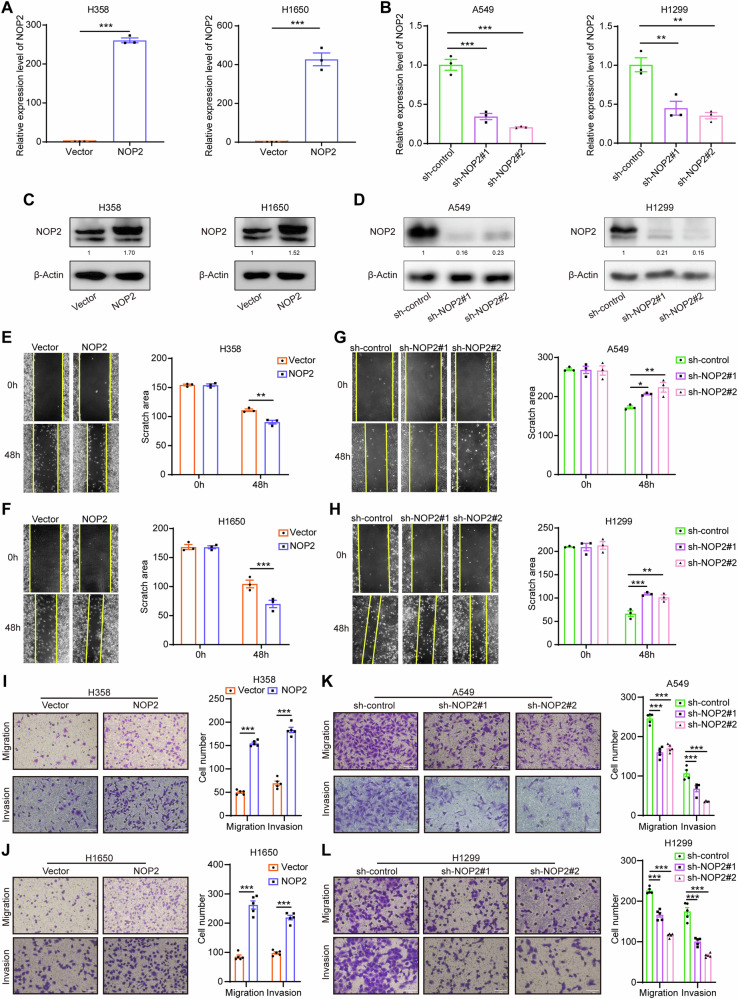
NOP2 promotes migration and invasion of lung cancer cells. **A**, **B** RT-qPCR was performed to verify the overexpression and knockdown efficiency of NOP2. **C**, **D** Western blot was performed to verify the overexpression and knockdown efficiency of NOP2. **E**–**H** The migration ability of lung cancer cells was determined by wound**-**healing assays. Scale bar = 100μm. **I**–**L** The invasion ability of lung cancer cells was shown by transwell assays. Scale bar = 100μm. Statistical methods: independent samples t-test (**A**, **E**, **F**, **I**, **J**), one-way ANOVA (**B**, **G**, **H**, **K**, **L**). Data are shown as the mean ± SEM of at least 3 independent experiments. *p < 0.05, **p < 0.01, ***p < 0.001.

Based on the aforementioned RT-qPCR and western blot findings, NOP2 expression was comparatively low in H358 and H1650 cells, but relatively high in A549 and H1299 cells. Consequently, to elucidate the potential role of NOP2 in lung cancer cells, we initially conducted overexpression of NOP2 in H358 and H1650 cells, and suppressed NOP2 in A549 and H1299 cells. The efficiency of NOP2 overexpression and knockdown was subsequently confirmed through RT‐qPCR and western blot analysis. The results demonstrated a significant alteration in NOP2 expression in lung cancer cells following the aforementioned treatments (Fig. 2A–D). Subsequently, on this basis, we carried out wound**-**healing and transwell assays to measure the migration ability of lung cancer cells. We found that overexpression of NOP2 markedly promoted the migration ability of H358 and H1650 cells (Fig. 2E, F, I, J), whereas attenuation of NOP2 significantly inhibited the migration ability of A549 and H1299 cells (Fig. 2G, H, K, L). Furthermore, transwell matrix penetration assays were conducted utilizing cell invasion chambers to evaluate the effect of NOP2 on the invasion of lung cancer cells. Overexpression of NOP2 caused substantial augmentation of the invasion of lung cancer cells compared with the respective control groups. In contrast, upon knockdown of NOP2, a significant inhibition of the invasion of lung cancer cells was observed in comparison with the corresponding control groups. Overall, these findings imply that the expression of NOP2 exerts a positive influence on the advancement of lung cancer cells in vitro.

### NOP2 does not affect the proliferation, cell cycle, and apoptosis of lung cancer cells

**Fig. 3:**
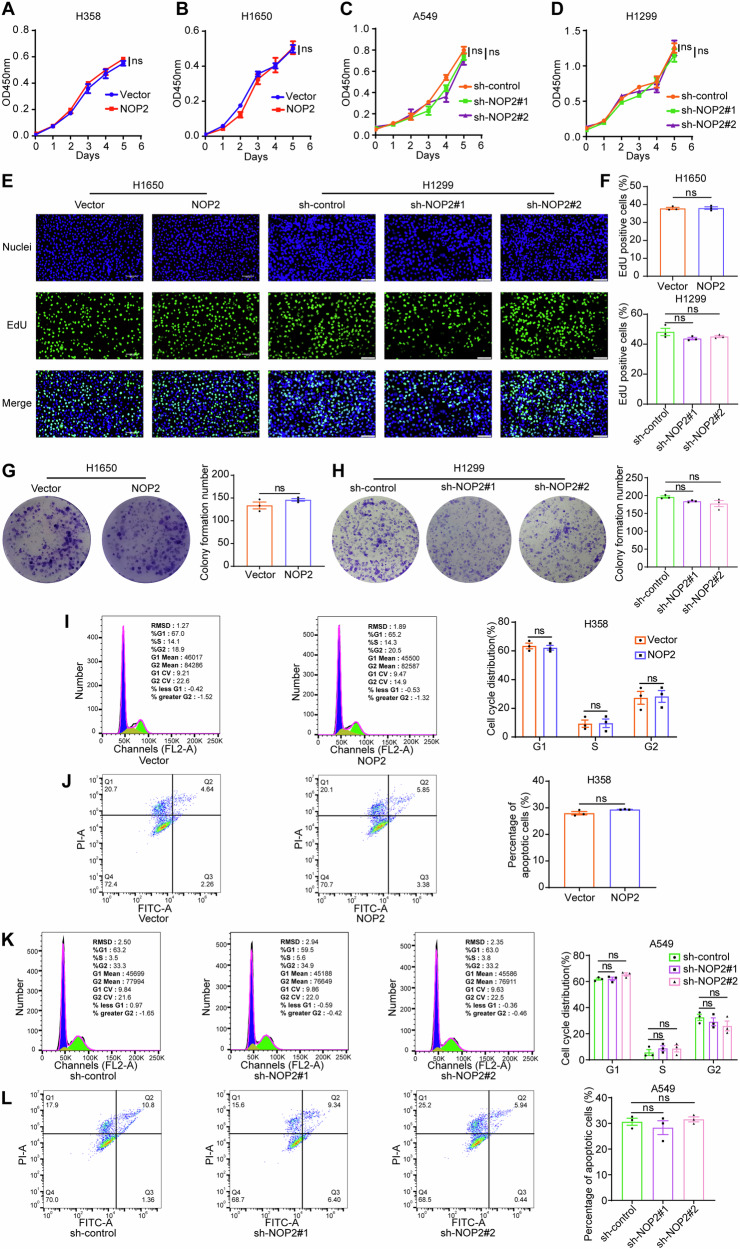
NOP2 does not affect the proliferation, cell cycle and apoptosis of lung cancer cells. **A**–**D** The effect of NOP2 on the proliferation of lung cancer cells was detected in CCK‐8 proliferation assay. **E**–**H** The effect of NOP2 on the proliferation of H1650 and H1299 cells was detected in EdU (Scale bar = 100μm) and colony-formation assays. **I**–**L** Cell cycle and apoptosis of H358 and A549 cells were detected by flow cytometry. Statistical methods: independent samples t-test (**A**, **B**, **F**, **G**, **I**, **J**), one-way ANOVA (**C**, **D**, **F**, **H**, **K**, **L**). Data are presented as the mean ± SEM of at least 3 independent experiments. *p < 0.05, **p < 0.01, ***p < 0.001.

To investigate whether NOP2 regulates cell proliferation of lung cancer cells, we conducted CCK-8, EdU, and colony-formation assays to determine the proliferation changes of lung cancer cells. The CCK-8 proliferation curves demonstrated that the manipulation of NOP2 expression, either through overexpression or knockdown, did not yield a statistically significant effect on the rate of cell proliferation compared with the control group (Fig. 3A–D). Similarly, the findings from the EdU and colony-formation assays indicated that alterations in NOP2 expression levels had no significant effect on the proliferation of H1650 and H1299 cells (Fig. 3E–H). This finding was further supported by the results obtained from H358 and A549 cells (Figure S1A–D). Subsequently, an examination of the cell cycle distribution was conducted. Flow cytometry analysis revealed that there was no statistically significant disparity in the proportion of S-phase cells that exhibited overexpression of NOP2 in H358 cells or knockdown of NOP2 in A549 cells, compared with their respective control groups (Fig. 3I, K). Subsequently, we assessed the effect of NOP2 on cell apoptosis. The findings obtained through Annexin V/PI double staining and flow cytometry in H358 and A549 cells demonstrated that the number of apoptotic cells following overexpression or knockdown of NOP2 did not significantly differ from that in the control group (Fig. 3J, L). The results of experiments to detect cell cycle distribution in H1650 and H1299 cells were consistent with the abovementioned results (Figure S1E, F). Thus, these results indicate that NOP2 has no significant effect on the proliferation, cell cycle, and apoptosis of lung cancer cells.

### EZH2 is a downstream gene of NOP2 in lung cancer cells

**Fig. 4:**
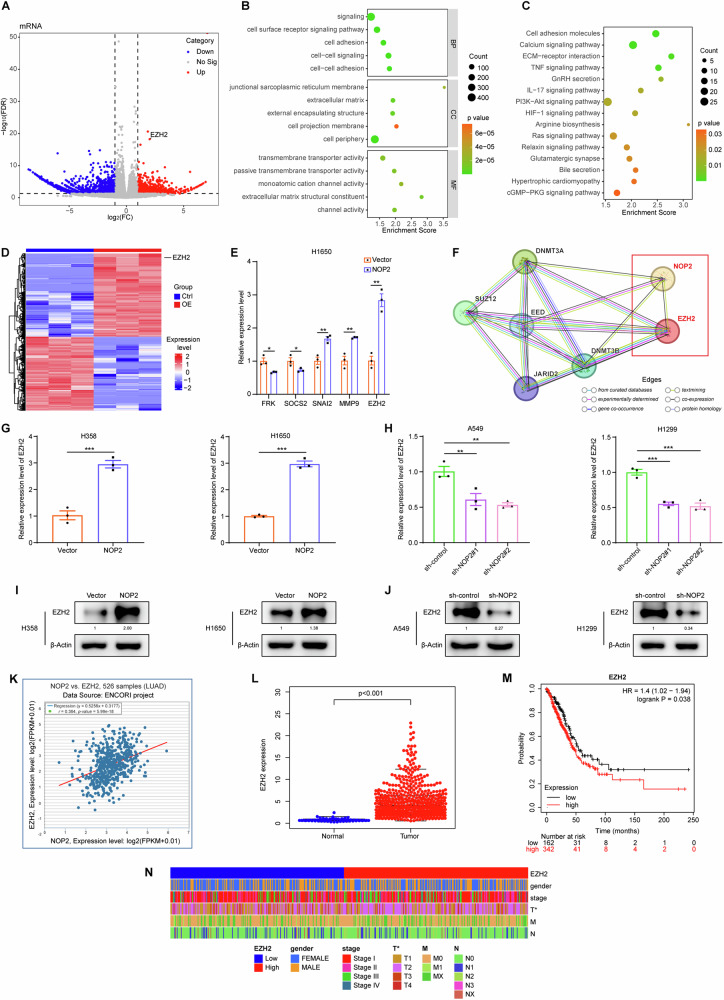
EZH2 is a downstream gene of NOP2 in lung cancer cells. **A**, **D** Volcano plot and heat map of RNA-sequencing showing the genes whose expression level was positively or negatively correlated with the expression level of NOP2. **B**, **C** GO and KEGG analysis for differential genes obtained by RNA-sequencing. **E** RT-qPCR was performed to detect mRNA levels of five differential genes associated with migration and invasion. **F** STRING database analysis of the protein interaction relationship. The types of connection represented by the different colored edges between the nodes are indicated in the figure. EZH2 expression upon NOP2 knockdown or overexpression were determined by **G**, **H** RT-qPCR and **I**, **J** western blot. **K** ENCORI database analysis of the correlation between NOP2 and EZH2 expression in LUAD. **L** Differential analysis of EZH2 expression in LUAD and normal tissues. **M** Kaplan–Meier analysis of EZH2. **N** The heat map of the relationship between EZH2 expression and different clinicopathologic features of LUAD. Statistical methods: independent samples *t*-test (**E**, **G**), one-way ANOVA (**H**). Data are shown as the mean ± SEM of at least 3 independent experiments. *p < 0.05, **p < 0.01, ***p < 0.001.

To investigate the molecular mechanism through which NOP2 influences the progression of lung cancer, we utilized RNA-sequencing analysis. The analysis involved the examination of differentially expressed genes (DEGs) in NOP2-overexpressing H1650 cells relative to control cells, with a twofold cutoff. The volcano plot of the up- and downregulated genes among the DEGs is presented in Fig. 4A. Furthermore, we conducted GO enrichment analysis (Fig. 4B), which revealed that DEGs were primarily associated with signaling, cell surface receptor signaling pathway, cell adhesion, and cell–cell signaling in terms of biological processes (BPs). For the cellular component (CC) functional category, enrichment of DEGs was mainly observed on the junctional sarcoplasmic reticulum membrane, extracellular matrix and external encapsulating structure. Additionally, within the molecular function (MF) functional category, DEGs showed enhancement in transmembrane transporter activity and passive transmembrane transporter activity. KEGG analysis (Fig. 4C) revealed that the primary KEGG pathways comprised cell adhesion molecules, calcium signaling pathway, and ECM–receptor interaction. In addition, the heat map of the up- and downregulated genes among the DEGs is presented in Fig. 4D. We identified and selected five genes associated with migration and invasion. RT-qPCR was utilized to identify the presence of these five genes in the original samples. Among these genes, EZH2 exhibited the most pronounced increase following overexpression of NOP2 (Fig. 4E). Subsequently, we investigated the potential co-expression relationship between NOP2 and EZH2 using the STRING database (Fig. 4F).

Therefore, our hypothesis posits EZH2 as a potential target of NOP2 in lung cancer cells, with NOP2 promoting lung cancer progression through the regulation of EZH2 expression. To validate this hypothesis, we assessed the EZH2 mRNA expression in lung cancer cells using RT-qPCR. The results indicated an increase in EZH2 mRNA expression upon NOP2 overexpression (Fig. 4G) and a significant decrease when NOP2 was knocked down (Fig. 4H). Furthermore, western blot results confirmed an elevation in EZH2 protein expression with NOP2 overexpression and a reduction upon NOP2 knockdown (Fig. 4I, J). To further validate our conjecture, we performed relevant bioinformatics analysis. Based on the ENCORI database, the expression levels of EZH2 and NOP2 were observably positively correlated in LUAD (*r* = 0.364, *P* = 5.99e-18, Fig. 4K). Analysis of EZH2 using the TCGA database confirmed that EZH2 was also highly expressed in LUAD tissues and correlated with poor prognosis and tumor stage (Fig. 4L–N). This result further supports our conjecture that EZH2 is the potential target of NOP2 in lung cancer cells.

### NOP2 promotes EZH2 mRNA stability in an m5C-dependent manner

**Fig. 5:**
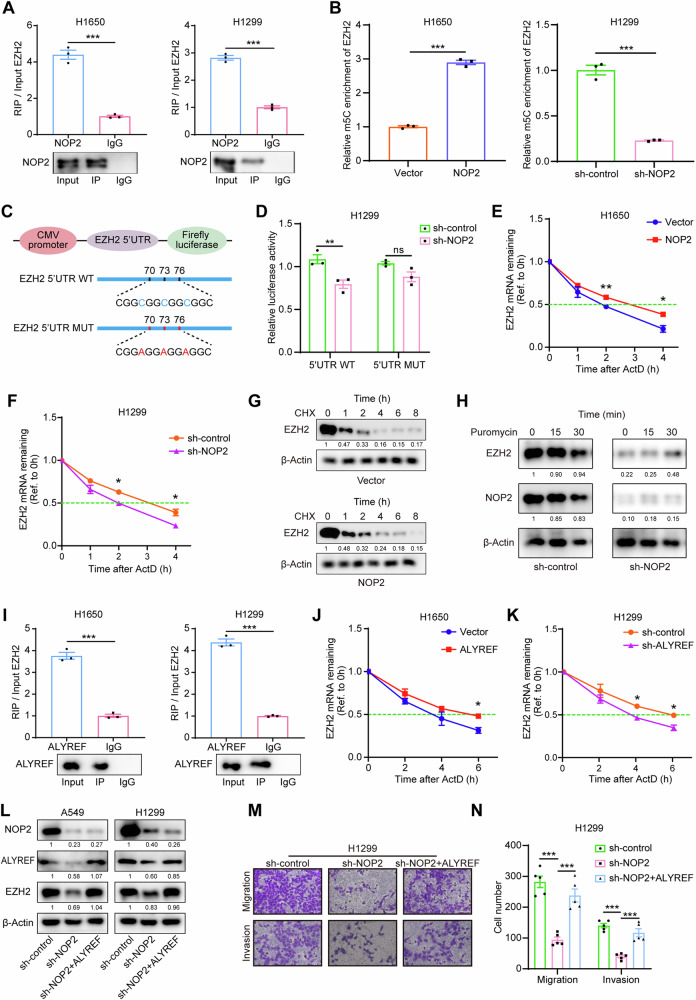
“Writer” NOP2 and “Reader” ALYREF promote the stability of EZH2 mRNA via m5C. **A**, **I** The results of RIP and RT-qPCR in H1650 and H1299 cells. IgG was used as a negative control to preclude nonspecific binding. **B** The results of methylated-RIP for H1650 and H1299 cells. The relative m5 C enrichment of EZH2 mRNA for each group was normalized to the Input. **C** EZH2 5’UTR containing either wild-type or mutant (C-to-A mutation) m5C sites was cloned into luciferase reporter vector. **D** Relative luciferase activity of the wild-type and mutant form of EZH2 5’UTR reporter vectors in H1299 cells transfected with sh-control or sh-NOP2, respectively. **E**, **F** The relative expression of EZH2 mRNA was detected after treating H1650 and H1299 cells with 5 μg/mL actinomycin D for indicated times. **J**, **K** Do the same. **G** H1299 cells in the control group and NOP2 overexpression group were treated with 100 μg/mL CHX for the indicated times, and protein expression of EZH2 was analyzed by western blot analysis. **H** H1299 cells transfected with sh-control or sh-NOP2 were treated with 200 ng/mL puromycin for the indicated time and the whole cell lysates were detected by western blot. **L** The expression level of EZH2 protein in A549 and H1299 cells of each group. **M**, **N** The results of the transwell assays. Scale bar = 100μm. Statistical methods: independent samples t-test (**A, B, D, E, F, I**, **J**, **K**), one-way ANOVA (**N**). Data are presented as the mean ± SEM of at least 3 independent experiments. *p < 0.05, **p < 0.01, ***p < 0.001.

To investigate whether NOP2 influences EZH2 expression through direct or indirect mechanisms, we conducted an RNA immunoprecipitation (RIP) assay to determine whether NOP2 protein could bind to EZH2 mRNA. Utilizing magnetic beads and antibodies against NOP2, we isolated the RNAs capable of interacting with NOP2 protein from H1650 and H1299 cells, and subsequently detected these RNAs using RT-qPCR. The findings revealed the inclusion of EZH2 mRNA in the RNAs isolated from H1650 and H1299 cells (Fig. 5A), indicating that NOP2 protein could directly bind to EZH2 mRNA. Overall, these results suggest that NOP2 protein can directly target EZH2 mRNA and influence the expression level of EZH2. To validate whether NOP2 regulates the m5C level of EZH2 mRNA, we conducted a methylated-RIP assay using stably transfected H1650 and H1299 cells. By employing magnetic beads and an m5C antibody, we isolated RNAs and subsequently analyzed these RNAs through RT-qPCR. Elevated levels of an mRNA in the isolated RNAs corresponded to higher m5C levels of the mRNA. The results of the m5C methylation RIP indicated that overexpression of NOP2 enhanced the m5C level of EZH2 mRNA in H1650 cells, while the knockdown of NOP2 reduced the m5C level of EZH2 mRNA in H1299 cells (Fig. 5B).

To further ascertain the role of NOP2 in controlling the m5C modification of EZH2, we constructed wild-type EZH2 (5’UTR WT) and mutant EZH2 (5’UTR MUT) luciferase reporter plasmids (Fig. 5C), and the luciferase activity of the wild-type or mutant EZH2-fused reporter was measured in control and NOP2-knockdown H1299 cells. As anticipated, knockdown of NOP2 significantly decreased luciferase activity in the EZH2 5’UTR WT group, but had no significant effect on the EZH2 5’UTR MUT group (Fig. 5D). These findings suggest that NOP2 may regulate EZH2 expression levels in an m5C-dependent manner. To elucidate the mechanism by which NOP2-mediated m5C regulates the expression of EZH2 mRNA, we investigated the stability of EZH2 mRNA in H1650 and H1299 cells treated with actinomycin D (ActD, 5 µg/mL). Following ActD treatment for 0, 1, 2, or 4 h, RNA was extracted and subjected to RT-qPCR analysis. The stability of EZH2 mRNA was determined by the degradation rate, normalized to the expression at 0 h. As depicted in Fig. 5E,F, a higher m5C level of EZH2 mRNA correlated with a longer time to decay to 50%, indicating better stability. In other words, NOP2 could enhance the stability of EZH2 mRNA in lung cancer cells through m5C modification. In addition, we explored whether NOP2 is involved in protein stability and translational regulation of EZH2. H1650 cells were treated with the protein-translation inhibitor cycloheximide (CHX), and western blot analysis showed that the half-life of EZH2 protein was similar in the control and NOP2-overexpression groups (Fig. 5G). H1299 cells were treated with puromycin for the indicated time periods, and the results from western blot analysis showed no significant difference in the translation efficiency of EZH2 protein between the control group and the NOP2-knockdown group (Fig. 5H). These results suggest that NOP2-induced EZH2 expression is associated with mRNA stability, but not with protein stability or translation.

### NOP2 regulates m5C modification of EZH2 via the m5C reader ALYREF

ALYREF, an important m5C reader, has been proven to maintain mRNA stability [20–22]. To verify whether ALYREF is a functional m5C reader in lung cancer cells, we performed RIP assay with ALYREF antibody in H1650 and H1299 cells, and the RT-qPCR results showed that ALYREF could bind to EZH2 mRNA, suggesting that ALYREF might be the m5C reader (Fig. 5I). Further research demonstrated that ALYREF expression affected the stability of EZH2 mRNA in H1650 and H1299 cells (Fig. 5J, K). To further unveil whether the tumor-promoting effects of NOP2 were dependent on ALYREF, we performed western blot and transwell assays. The results showed that the decrease in EZH2 expression triggered by NOP2 knockdown as well as the antitumor phenotype of lung cancer cells were suppressed by overexpression of ALYREF (Fig. 5L–N). In contrast, knockdown of ALYREF inhibited the elevation of EZH2 expression triggered by NOP2 overexpression as well as the enhancement of the migratory invasive ability of lung cancer cells (Fig. S2A–E). In addition, we also overexpressed EZH2 and knocked down ALYREF in A549 and H1299 cells. The results of transwell assay similarly demonstrated the important effects of ALYREF and EZH2 on the migration and invasion ability of lung cancer cells (Fig. S2F–I). Taking all the above results into consideration, we could draw such a conclusion that NOP2 could promote the expression of EZH2 by upregulating the stability of EZH2 mRNA in an m5C-dependent manner in lung cancer cells.

### NOP2 promotes EMT via regulating EZH2 in lung cancer cells

**Fig. 6:**
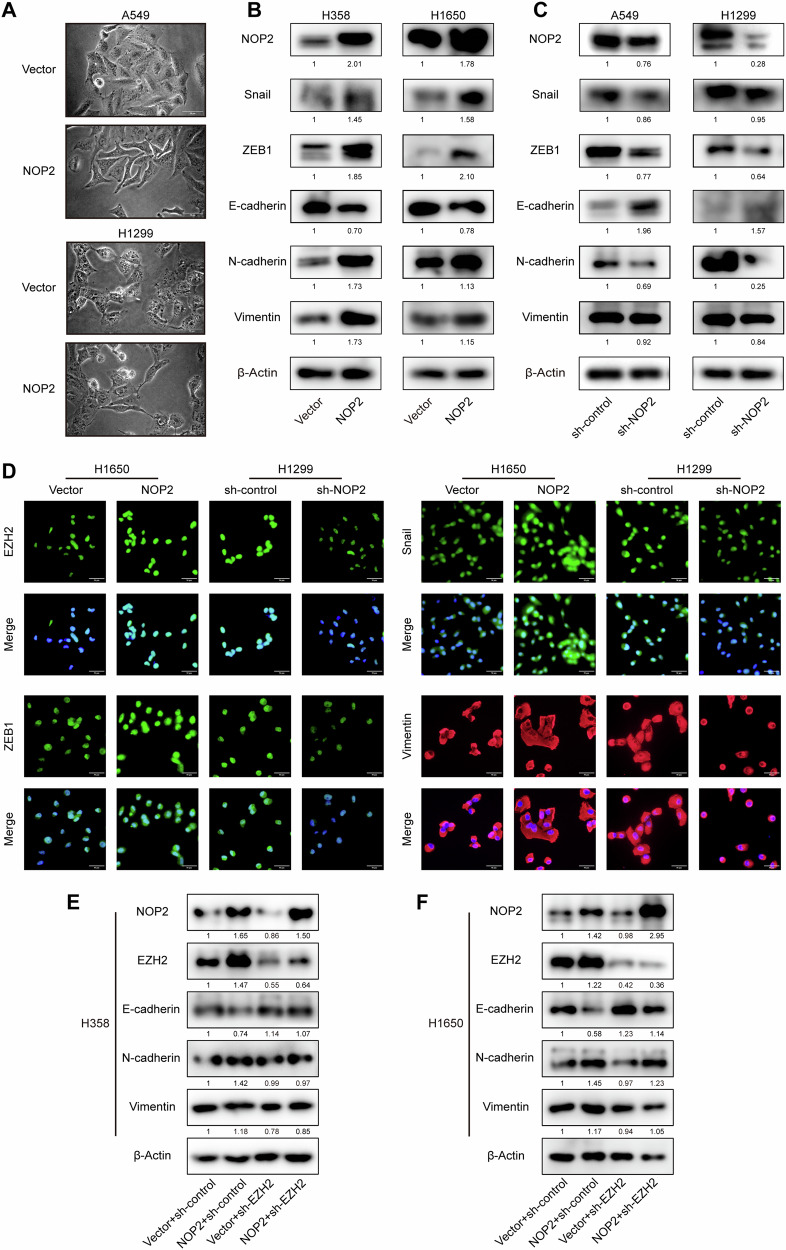
NOP2 promotes EMT via regulating EZH2 in lung cancer cells. **A** The morphology of A549 and H1299 cells was observed using a microscope. Scale bar = 50 μm. **B**, **C** Protein expression levels in lung cancer cells were detected by western blot. **D** The effect of NOP2 on the expression of different molecules in H1650 and H1299 cells was detected by immunofluorescence. Scale bar = 50 μm. **E**, **F** The expression levels of the relevant proteins in H358 and H1650 cells were detected by western blot.

By integrating the findings from the prior experiments, which demonstrated the predominant impact of NOP2 on the migration and invasion of lung cancer cells, alongside the recognition of EZH2 as a crucial protein involved in the regulation of EMT, we postulated that NOP2 might also facilitate EMT in lung cancer cells. Initially, we observed that A549 and H1299 cells in the NOP2-overexpression group exhibited larger dimensions, diminished polarity, spindle-like morphology, and reduced intercellular junctions compared with cells in the control group (Fig. 6A). Following this, we conducted an analysis of the relationship between NOP2 and EMT-related genes in lung cancer. Our findings revealed a negative correlation between NOP2 and CDH1 expression, while the expression levels of CDH2 and SNAI1 exhibited positive correlations with NOP2 (Fig. S2K–M). On this basis, we detected the expression levels of EMT-related markers by western blot assays. The results showed that following overexpression of NOP2, the expression of E-cadherin was decreased, and the expression levels of Snail, ZEB1, N-cadherin, and vimentin were increased; the opposite was true following NOP2 knockdown (Fig. 6B, C). Next, we performed cellular immunofluorescence (IF) experiments and similarly demonstrated the promotional effect of NOP2 for EMT in lung cancer cells (Fig. 6D, Fig. S3A).

**Fig. 7:**
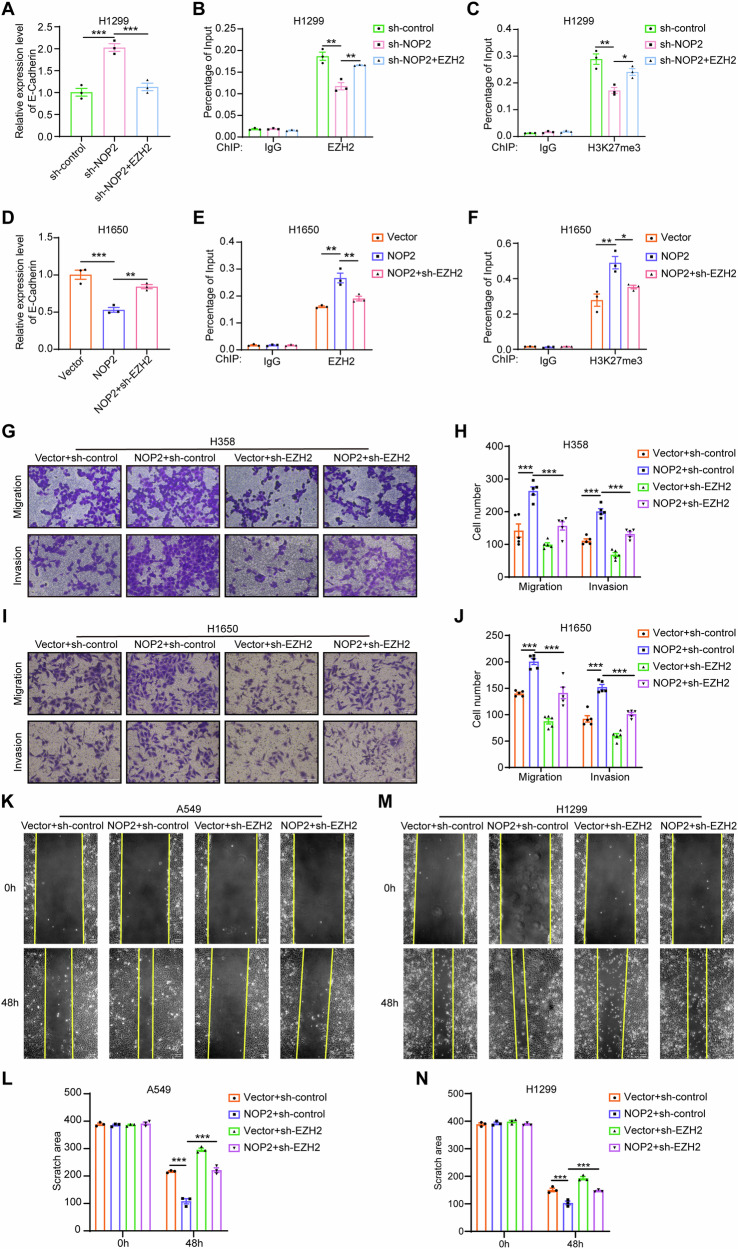
EZH2 deficiency can counterbalance the positive effect of NOP2 on lung cancer cells in vitro. **A** RT-qPCR was performed to detect the expression level of E-cadherin mRNA in each group of H1299 cells. **B**, **C** Binding of EZH2 and H3K27me3 antibodies to the E-cadherin promoter was detected by ChIP assay in H1299 cells. **D**–**F** Set up opposite NOP2 and EZH2 expression groups and do the same experiments in H1650 cells. Transwell (**G**–**J**) and wound-healing assays (**K**–**N**) were performed to detect the migratory and invasive abilities of lung cancer cells. Scale bar = 100 μm. Statistical methods: one-way ANOVA (**A**–**F**, **H**, **J**, **L**, **N**). Data are presented as the mean ± SEM of at least 3 independent experiments. *p < 0.05, **p < 0.01, ***p < 0.001.

To further explore the mechanism by which NOP2 promotes EMT, we knocked down EZH2 in H358 cells and H1650 cells overexpressing NOP2 and examined the expression levels of EMT-related proteins (Fig. 6E, F). The results showed that deletion of EZH2 attenuated the effect of NOP2 on the related proteins. This suggests that EZH2 plays a key role in the promotion of EMT by NOP2. EZH2 expression has been found to catalyze histone H3K27 trimethylation (H3K27me3) at the promoter of the epithelial gene E-cadherin, leading to silencing of E-cadherin, which promotes cellular EMT development and tumor invasion [23, 24]. Thus, we examined the mRNA expression of E-cadherin in H1299 cells. RT-qPCR results showed that the expression of E-cadherin was significantly upregulated in the NOP2-knockdown group, whereas overexpression of EZH2 was able to counteract the effect of NOP2 knockdown on the expression level of E-cadherin (Fig. 7A). Correspondingly, we performed chromatin immunoprecipitation (ChIP) experiments using EZH2 antibody and H3K27me3 antibody. ChIP-qPCR analysis confirmed that depletion of NOP2 led to a reduction in the binding capacity of EZH2 and H3K27me3 to the promoter regions of E-cadherin, whereas overexpression of EZH2 prevented this effect (Fig. 7B, C). Similarly, overexpression of NOP2 and knockdown of EZH2 in H1650 cells led to the opposite effect (Fig. 7D–F). Overall, NOP2 altered the expression of EZH2’s downstream target E-cadherin by regulating EZH2 expression and H3K27me3 mediated by EZH2, and thus promoted EMT in lung cancer cells.

### EZH2 deficiency can counterbalance the positive effect of NOP2 on lung cancer cells in vitro

We next performed transwell and wound**-**healing assays to validate the critical mediator role of EZH2. As indicated by the results of transwell assays, overexpression of NOP2 enhanced the migration and invasion capabilities of H358 and H1650 cells, but this promotional effect was mitigated by the removal of EZH2. In addition, downregulation of EZH2 inhibited the migration and invasion of H358 and H1650 cells (Fig. 7G–J). Similar findings were observed in wound-healing assays (Figs. 7K–N and S3B–E). Collectively, these results demonstrate that NOP2 promotes lung cancer progression by upregulating EZH2 expression, and this promoting effect can be offset by the absence of EZH2.

### EZH2 deficiency represses the positive effect of NOP2 on lung cancer progression in vivo

**Fig. 8:**
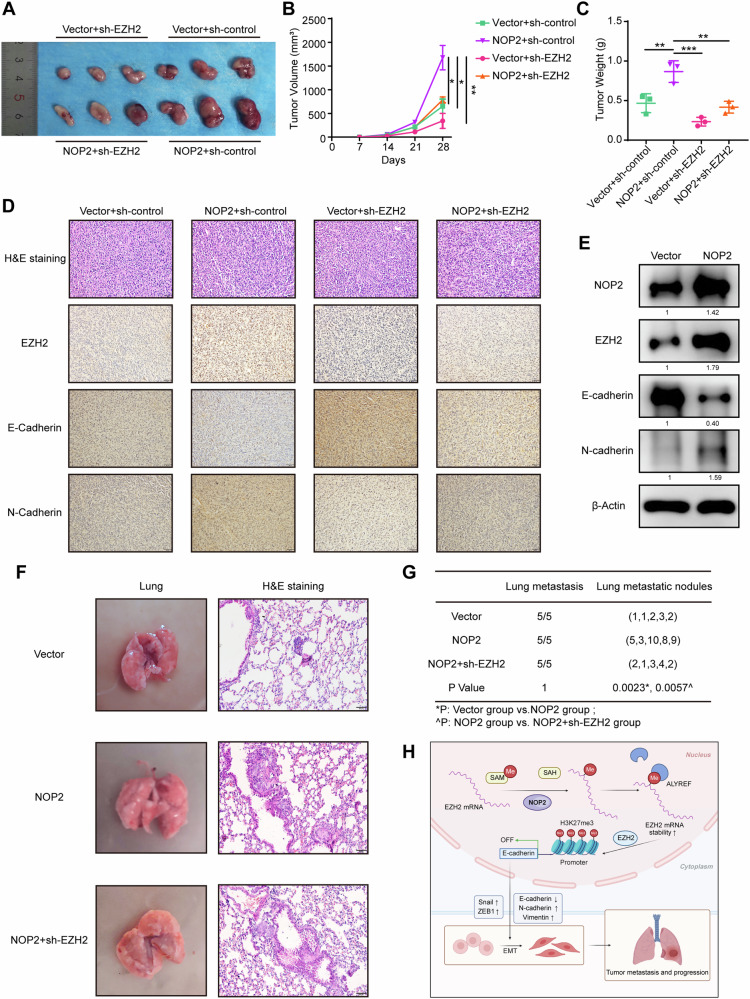
EZH2 deficiency represses the positive effect of NOP2 on lung cancer progression in vivo. **A**–**C** Tumor growth and weight were monitored in mice. **D**, **E** IHC staining of tumor sections and western blot results of tumor proteins. Scale bar = 50 μm. **F** Images of lungs in an in vivo mouse metastatic tumor model and H&E staining images of lung sections. Scale bar = 100 μm. **G** Lung metastases in all mice and the number of nodules were counted. **H** Schematic summarizing the NOP2/EZH2/EMT axis and its role in modulating lung cancer progression. Statistical methods: one-way ANOVA (**B**, **C**, **G**). Data are presented as the mean ± SEM of at least 3 independent experiments. *p < 0.05, **p < 0.01, ***p < 0.001.

To validate the functional role of the NOP2–EZH2 axis in lung cancer progression, animal models were used to further verify the function of this axis in vivo. Tumor xenografts were established in immunodeficient nude mice using stably transfected H1299 cells. The growth of the tumors was monitored and recorded, and upon completion of the study, all nude mice were euthanized and the tumors were weighed (Fig. 8A–C). As anticipated, the overexpression of NOP2 facilitated the in vivo growth of lung cancer cells, whereas the introduction of sh-EZH2 mitigated this promoting effect. The IHC results (Fig. 8D) further confirmed that NOP2 overexpression led to a downregulation of E-cadherin expression, which was counteracted by the introduction of sh-EZH2. Additionally, the western blot analysis of tumor proteins yielded consistent results with the aforementioned findings (Fig. 8E). Next, a hematogenous metastasis model by tail vein injection was established to validate the effect of NOP2 on metastasis in vivo. Remarkably, although lung metastasis occurred in all of the mice, the lung metastatic foci in the NOP2-overexpression group were significantly larger and more numerous than those in the control vector group (Fig. 8F–G), whereas the lung metastasis in the EZH2-knockdown group was similar to that in the control vector group. Taken together, these findings suggest that NOP2 can promote the development of lung cancer in vivo by regulating EZH2.

## Discussion

Lung cancer remains a great challenge to mankind due to its high incidence, and aggressiveness, and poor prognosis. Accumulating evidence has indicated that m5C methylation plays a critical role in the progression of human diseases, including cancer [25–27]. However, to date, research on m5C modification and its roles in lung cancer remains limited.

In this study, we observed an upregulation of NOP2 in lung cancer tissues compared with normal tissues. The heightened expression of NOP2 was associated with unfavorable prognosis, consistent with prior research [19, 28]. Functional experiments showed that NOP2 promoted migration and invasion of lung cancer cells in vitro, thereby enhancing tumor growth and progression in vivo. It has been reported that NOP2 can be regulated by the long noncoding RNA, LINC00963, to promote metastasis of prostate cancer cells [29]. In addition, Yang et al.’s research showed that NOP2 promotes the development and progression of high-grade serous ovarian cancer by regulating Rap guanine nucleotide exchange factor 4 (RAPGEF4) [30]. Notably, NOP2 facilitated the proliferation of ovarian cancer cells in vitro and in vivo in this study. Another study found the promotional effect of NOP2 on the proliferation of gastric adenocarcinoma cells [31]. However, in our study, NOP2 did not affect the proliferative capacity of lung cancer cells. The observed difference may be related to different histological types. Additionally, we observed a promotional effect of NOP2 on tumor growth in nude mouse xenograft models. This disparity likely stems from the intricate complexity of the in vivo environment, encompassing factors such as intercellular interactions, immune system mediation, the diversity of metabolic and signaling pathways, as well as the dynamic nature of epigenetic modifications. Consequently, it is of paramount importance to validate efficacy and safety through animal models and clinical trials before translating laboratory findings into clinical applications. Altogether, our findings further confirm the oncogenic role of NOP2 in lung cancer.

To delve deeper into the regulatory mechanisms underlying lung cancer progression, we identified EZH2 as a downstream target of NOP2 through RNA-sequencing and bioinformatics analysis. EZH2, a histone methyltransferase subunit of a Polycomb repressor complex, is upregulated in a variety of human malignancies, such as prostate, breast, and gastric cancers [32–34]. Accumulating data show that EZH2 is involved in some tumor-related biological processes, including cell proliferation, autophagy and apoptosis, metastasis, and immunomodulation [35–40]. Importantly, Liu and colleagues identified that EZH2 could play a tumorigenic role in lung cancer [41]. Similarly, we found that EZH2 positively correlated with NOP2, and EZH2 silencing partially reversed the oncogenic effects of NOP2 overexpression. Furthermore, EZH2 could potentially mediate the metastatic effects induced by NOP2 by influencing the expression of EMT-related proteins. These discoveries unveil a novel regulatory pathway through which NOP2 exerts its oncogenic influence—at least in part by upregulating EZH2 expression in lung cancer.

The m5C modification of mRNAs is associated with various biological processes, such as mRNA stabilization and translation, splicing, and nucleoplasmic transport [42]. ALYREF, an important m5C “reader,” mainly affects the nuclear export and stability of target mRNAs [10, 43, 44]. A mechanistic study revealed that LINC02159 combined with ALYREF to enhance the stability of YAP1 mRNA through m5C modification, which led to the upregulation of YAP1 expression and activation of the Hippo and β-catenin signaling pathways, thereby promoting the progression of NSCLC [20]. Here, we found that NOP2 and ALYREF had a co-regulatory role in the stability of EZH2 mRNA in lung cancer. Notably, knockdown of NOP2 in A549 and H1299 cells appears to reduce ALYREF expression levels, whereas overexpression of NOP2 in H358 and H1650 cells has no significant effect on ALYREF expression. The regulatory relationship between NOP2 and ALYREF remains unexplored. A study uncovered an m5C-dependent cross-regulation between NSUN2 and ALYREF, which activates hypermethylated m5C oncogenic RNA by promoting splicing and maintaining stabilization, leading to the progression of uroepithelial bladder cancer [45]. As a member of the NSUN2 homologous family, we hypothesized that NOP2 may also interact with ALYREF. This was supported by protein interaction analysis (Fig. S2J). Further research is warranted to elucidate the relationship between NOP2 and ALYREF. In addition, most of the early studies on the role of NOP2 have focused on ribosomal RNA. A recent study has revealed that NOP2 regulates c-Myc mRNA stability and translation in an m5C-modified manner to promote glycolysis and hepatocellular carcinoma (HCC) progression [46]. In contrast, a study by Sun et al. showed that NOP2 suppressed the malignant phenotypes of HCC cells by enhancing m5C methylation of XPD [47]. It is evident that the role played by NOP2 in different diseases still requires more in-depth studies. Moreover, whether additional readers or m5C-related enzymes contribute to the regulation of EZH2 expression requires further exploration.

In summary, our investigation disclosed the upregulation of NOP2 in lung cancer, correlating it with unfavorable prognosis. Notably, we present novel evidence that NOP2 promotes lung cancer progression by enhancing EZH2 expression through an m5C-dependent mechanism. The regulatory mechanism is illustrated in Fig. 8H. Consequently, NOP2 may represent a promising therapeutic target for lung cancer treatment.

### Supplementary information

Supplementary Figures and Tables

Original western blots

## Data Availability

The data that support the findings of this study are available from the corresponding author upon reasonable request. The RNA-sequencing data have been deposited in the NCBI Gene Expression Omnibus under accession number GSE270282.
