# Supplementary material for: NOP2 facilitates EZH2-mediated epithelial–mesenchymal transition by enhancing EZH2 mRNA stability via m5C methylation in lung cancer progression
Source: Cell Death Dis. 2024 Jul 16;15(7):506. doi: 10.1038/s41419-024-06899-w (PMC11252406; doi:10.1038/s41419-024-06899-w)
Supplement: Supplementary file 1 — Supplementary Figures and Tables [file 41419_2024_6899_MOESM1_ESM.pdf]

Supplementary Figures and Tables

NOP2 facilitates EZH2-mediated epithelial–mesenchymal transition by enhancing EZH2 mRNA stability via m5C methylation in lung cancer progression

Ying Yang, Hongzhao Fan, Hongyang Liu, Xueling Lou, Nan Xiao, Chenxing Zhang, Huanxiang Chen, Shuangshuang Chen, Huihui Gu, Hongchun Liu, Junhu Wan

This PDF file includes:

Figure S1-S3

Table S1-S2

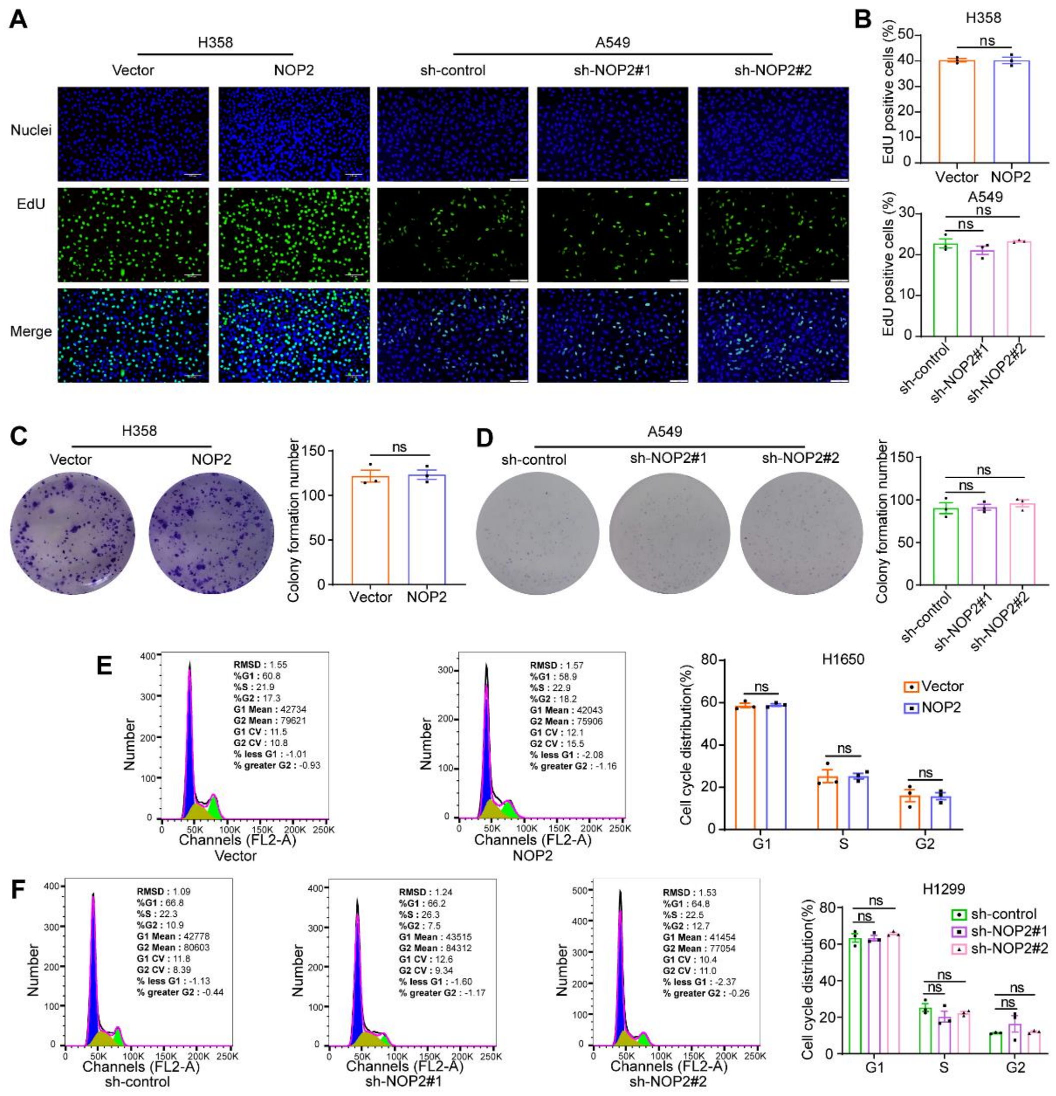

Figure S1 A-D The effect of NOP2 on the proliferation of H358 and A549 cells was detected in EdU (Scale bar = 100μm) and colony-formation assays. **E-F** Cell cycle of H1650 and H1299 cells were detected by flow cytometry. Statistical methods: independent samples t-test (**B, C, E**), one-way ANOVA (**B, D, F**). Data are presented as the mean ± SEM of at least 3 independent experiments. *$p < 0.05$, **$p < 0.01$, ***$p < 0.001$.

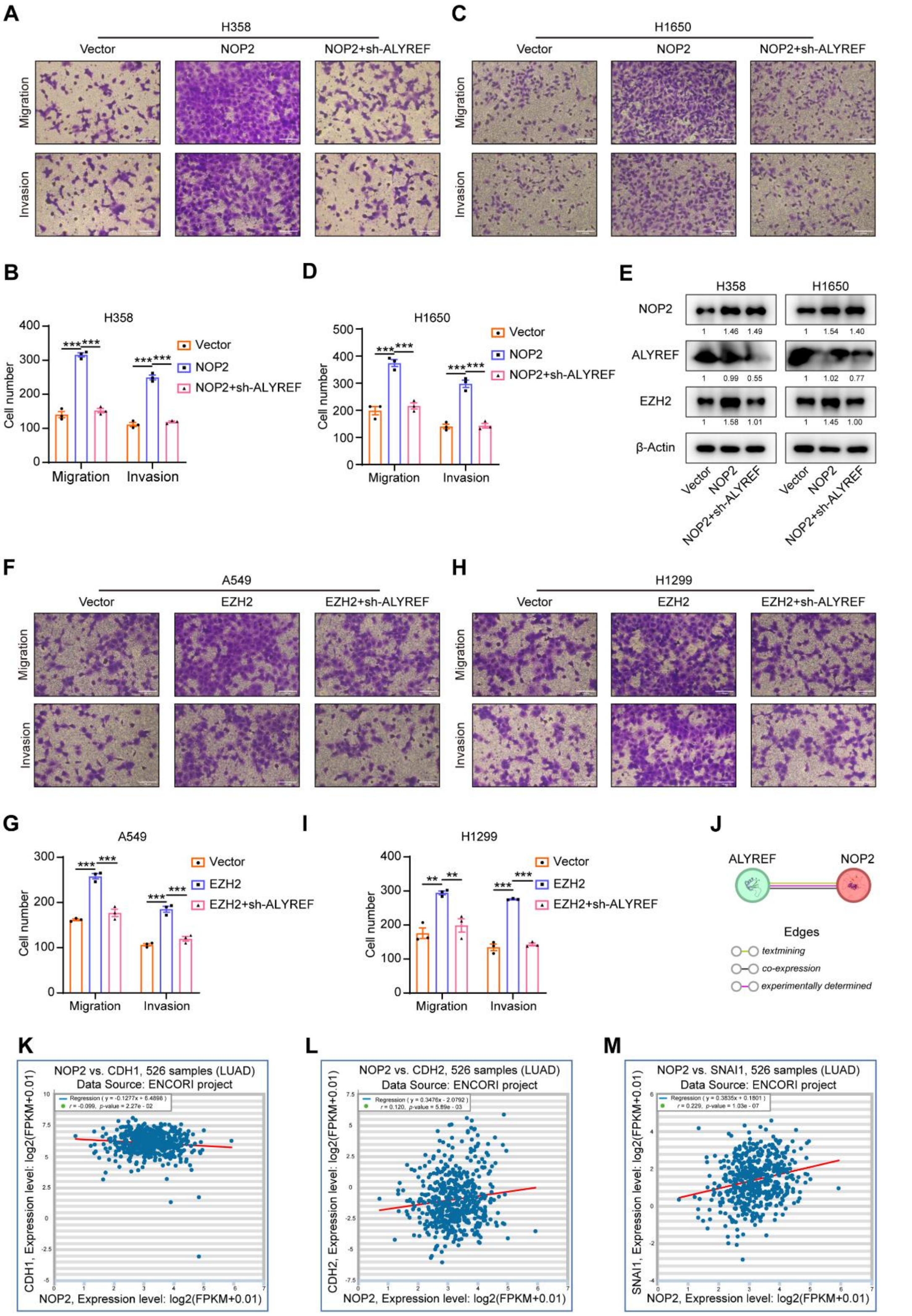

Figure S2 A-B The migration and invasion ability of each group in H358 cells was shown by transwell assays. **C-D** The migration and invasion ability of each group in H1650 cells was shown by transwell assays. **E** The expression level of relevant proteins

in H358 and H1650 cells of each group. **F-G** The migration and invasion ability of each group in A549 cells was shown by transwell assays. **H-I** The migration and invasion ability of each group in H1299 cells was shown by transwell assays. Scale bar = 100μm. **J** STRING database analysis of the protein interaction relationship. The types of connection represented by the different colored edges between the nodes are indicated in the figure. **K-M** ENCORI database analysis of NOP2 and CDH1, CDH2, SNAI1 expression correlation in LUAD. Statistical methods: one-way ANOVA (**B, D, G, I**). Data are presented as the mean ± SEM of at least 3 independent experiments. *$p < 0.05$, **$p < 0.01$, ***$p < 0.001$.

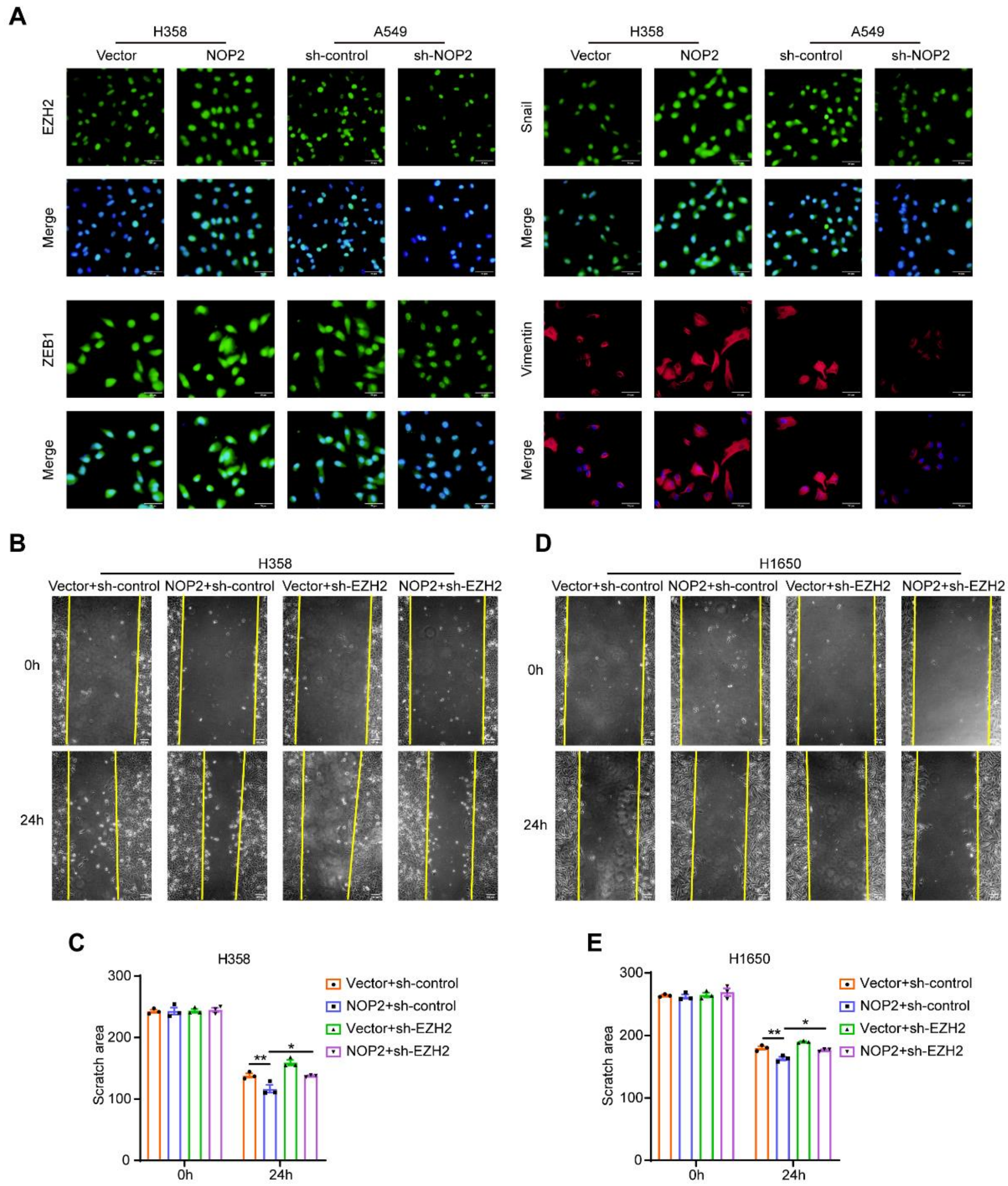

Figure S3 A The effect of NOP2 on the expression of different molecules in H358 and A549 cells was detected by immunofluorescence. Scale bar = 50μm. **B-E** Wound-healing assays were performed to detect the migratory of H358 and H1650 cells. Scale bar = 100μm. Statistical methods: one-way ANOVA (**C, E**). Data are presented as the mean ± SEM of at least 3 independent experiments. *$p < 0.05$, **$p < 0.01$, ***$p < 0.001$.

Table S1 Bioinformatics tools.

Bioinformatics tools and computer analysis software	Web site or title
TIMER 2.0 database	http://timer.comp-genomics.org
The Cancer Genome Atlas Program (TCGA)	https://portal.gdc.cancer.gov/
Data analysis software	R3.0.3、Perl
Kaplan-Meier Plotter database	http://kmplot.com/analysis/
STRING database	https://cn.string-db.org/
ENCORI database	https://rnasysu.com/encori/index.php

Table S2 List of primer sequences.

Gene	Sequence (5'-3')	Function
NOP2 Forward	AAGGGTGCCGAGACAGAACT	RT-qPCR
NOP2 Reverse	GAGCACGACTAGACAGCCTC	RT-qPCR
GAPDH Forward	GGAGCGAGATCCCTCCAAAAT	RT-qPCR
GAPDH Reverse	GGCTGTTGTCATACTTCTCATGG	RT-qPCR
EZH2 Forward	AGGACGGCTCCTCTAACCAT	RT-qPCR、RIP-qPCR
EZH2 Reverse	CTTGGTGTTGCACTGTGCTT	RT-qPCR、RIP-qPCR
FRK Forward	ACCGCAACTCCATACAGC	RT-qPCR
FRK Reverse	TTCCGAGACTCCAGATAGGC	RT-qPCR
SOCS2 Forward	TTAAAAGAGGCACCAGAAGGAAC	RT-qPCR
SOCS2 Reverse	AGTCGATCAGATGAACCACACT	RT-qPCR
SNAI2 Forward	CGAACTGGACACACATACAGTG	RT-qPCR
SNAI2 Reverse	CTGAGGATCTCTGGTTGTGGT	RT-qPCR
MMP9 Forward	AGTCCACCCTTGTGCTCTTCCC	RT-qPCR
MMP9 Reverse	TCTCTGCCACCCGAGTGTAACC	RT-qPCR
E-cadherin Forward	CAAGAACACACCATTGCACT	ChIP-qPCR
E-cadherin Reverse	TGCCAGTCTGTGCTAAGC	ChIP-qPCR
