# Supplementary figures and images for: NOP2 facilitates EZH2-mediated epithelial–mesenchymal transition by enhancing EZH2 mRNA stability via m5C methylation in lung cancer progression
Source: Cell Death Dis. 2024 Jul 16;15(7):506. doi: 10.1038/s41419-024-06899-w (PMC11252406; doi:10.1038/s41419-024-06899-w)

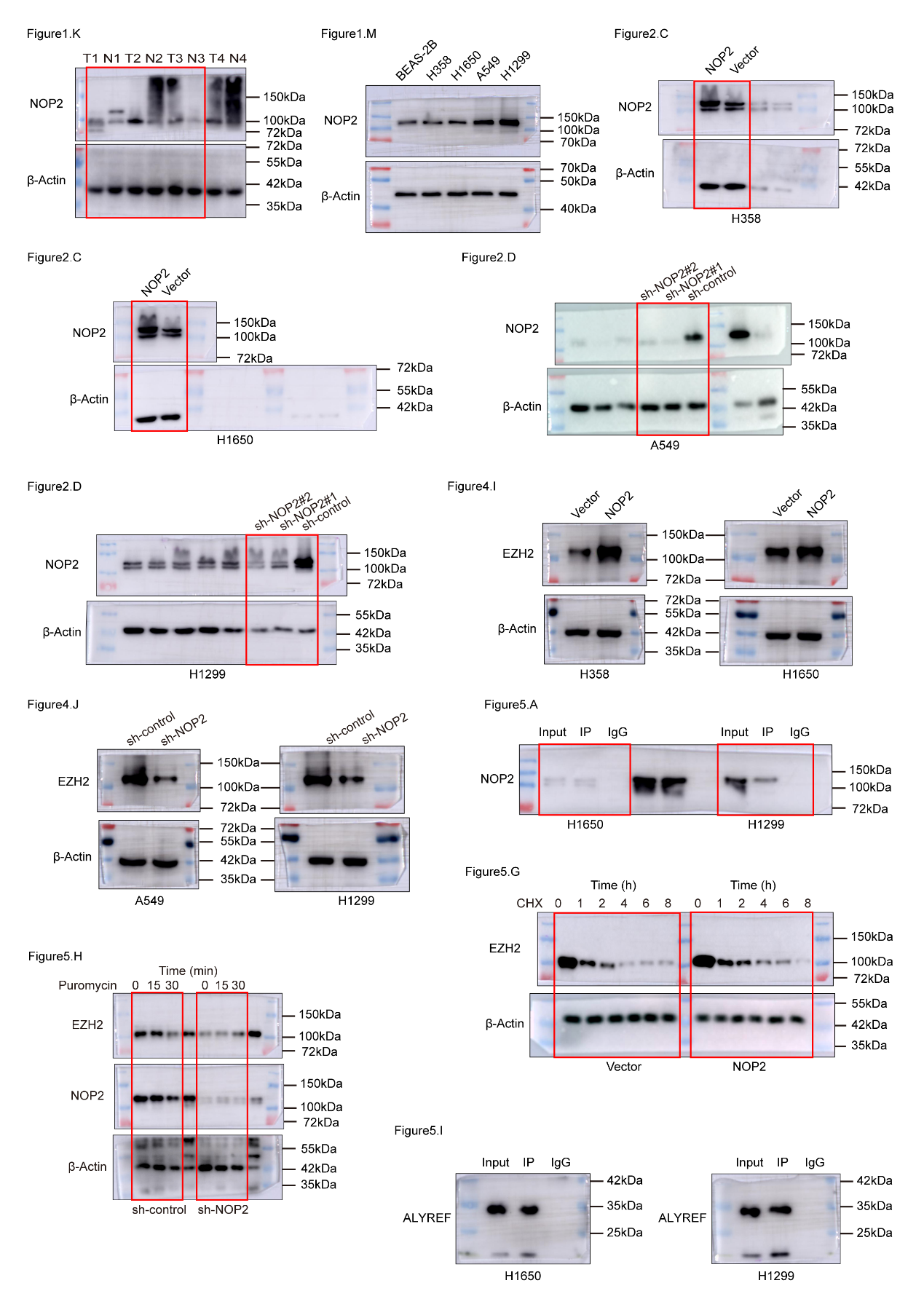


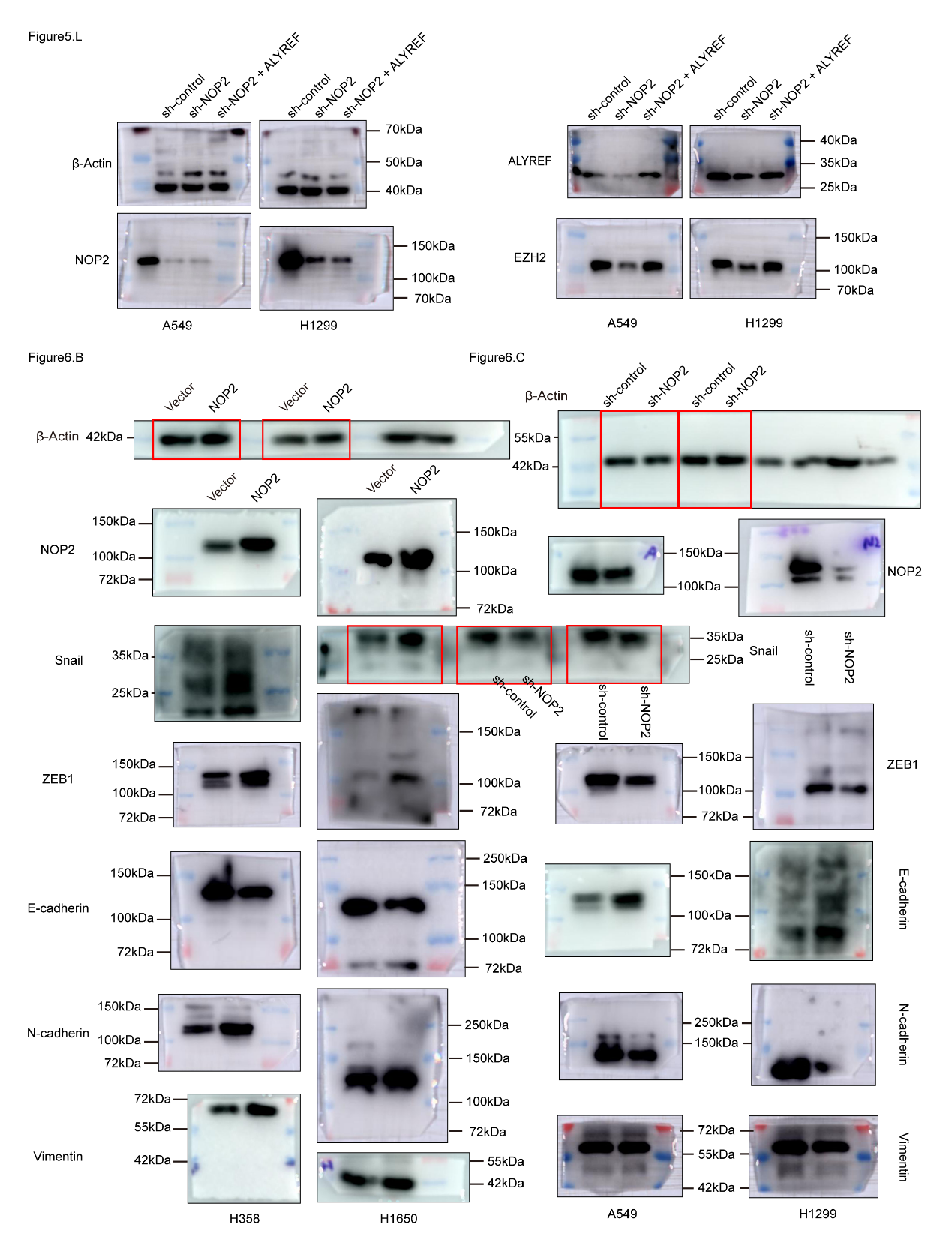


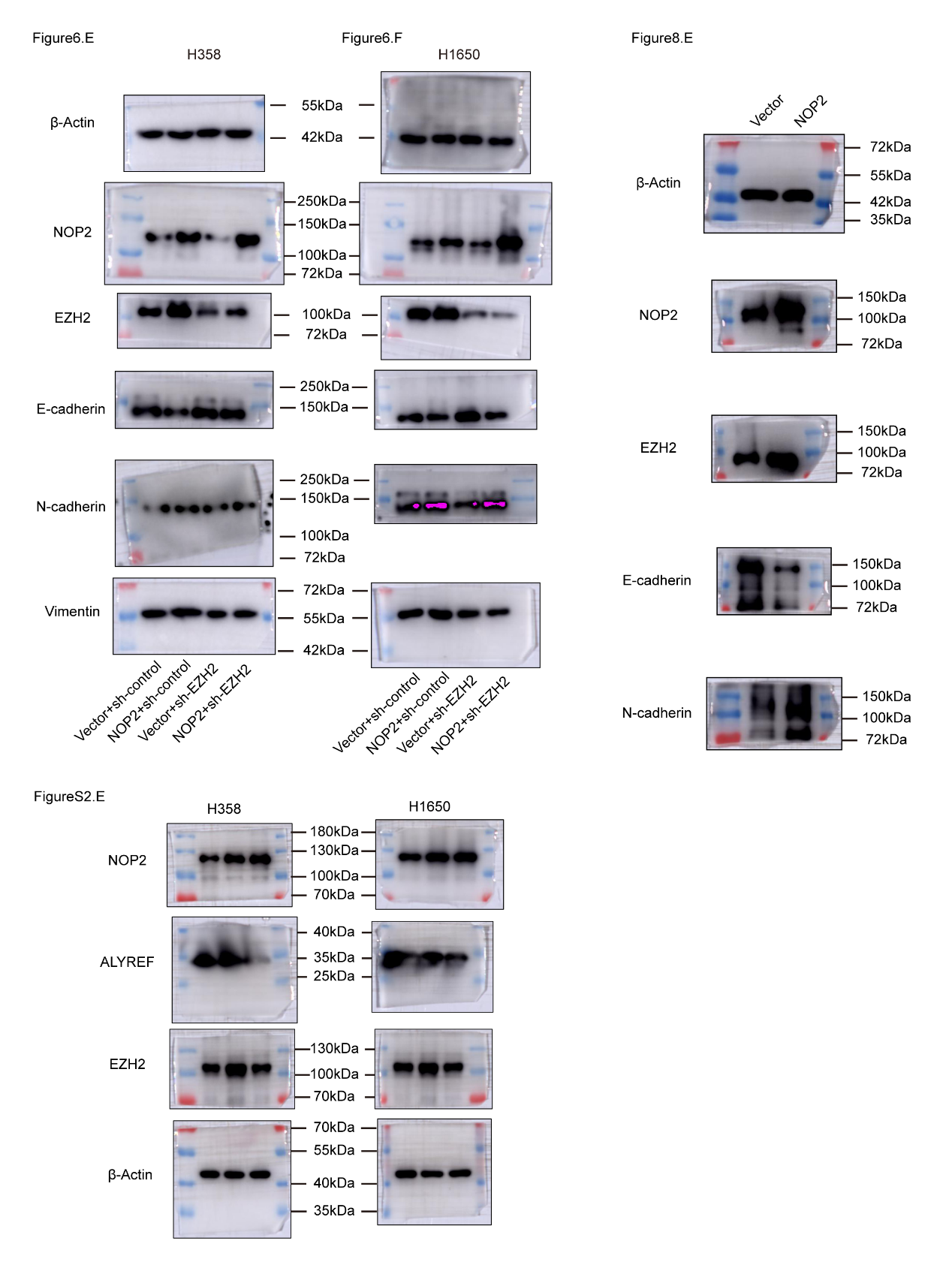

Supplement: Supplementary file 2 — Original western blots [file 41419_2024_6899_MOESM2_ESM.docx]
